# Can home care work be organized to promote health among the workers while maintaining productivity? An investigation into stakeholders’ perspectives on organizational work redesign concepts based on the Goldilocks Work principles

**DOI:** 10.1186/s12913-023-09691-2

**Published:** 2023-06-21

**Authors:** Ingeborg Frostad Liaset, Marius Steiro Fimland, Andreas Holtermann, Svend Erik Mathiassen, Skender Redzovic

**Affiliations:** 1 Department of Neuromedicine and Movement Science, Faculty of Medicine and Health Sciences, NTNU Norwegian University of Science and Technology, Trondheim, Norway; 2 Unicare Helsefort Rehabilitation Centre, Rissa, Norway; 3 National Research Centre for the Working Environment, Copenhagen, Denmark; 4 Department of Occupational Health Science and Psychology, Centre for Musculoskeletal Research, University of Gävle, Gävle, Sweden

**Keywords:** Job redesign, Participatory approach, Workplace health promotion, Home health care, Elder care

## Abstract

**Background:**

Due to the aging population, the need for home care services is increasing in most Western countries, including Norway. However, the highly physical nature of this job could contribute to make recruiting and retaining qualified home care workers (HCWs) challenging. This issue may be overcome by adopting the Goldilocks Work principles, aiming at promoting workers’ physical health by determining a “just right” balance between work demands and recovery periods while maintaining productivity. The aim of this study was to 1) gather suggestions from home care employees on suitable organizational (re)design concepts for promoting HCWs’ physical health and 2) have researchers and managers define actionable behavioral aims for the HCWs for each proposed (re)design concept and evaluate them in the context of the Goldilocks Work principles.

**Methods:**

HCWs, safety representatives, and operation coordinators (*n* = 14) from three Norwegian home care units participated in digital workshops led by a researcher. They suggested, ranked, and discussed redesign concepts aimed at promoting HCWs’ health. The redesign concepts were subsequently operationalized and evaluated by three researchers and three home care managers.

**Results:**

Workshop participants suggested five redesign concepts, namely "operation coordinators should distribute work lists with different occupational physical activity demands more evenly between HCWs", "operation coordinators should distribute transportation modes more evenly between HCWs", "Managers should facilitate correct use of ergonomic aids and techniques", "HCWs should use the stairs instead of the elevator", and "HCWs should participate in home-based exercise training with clients". Only the first two redesign concepts were considered to be aligned with the Goldilocks Work principles. A corresponding behavioral aim for a “just right” workload was defined: reduce inter-individual differences in occupational physical activity throughout a work week.

**Conclusions:**

Operation coordinators could have a key role in health-promoting organizational work redesign based on the Goldilocks Work principles in home care. By reducing the inter-individual differences in occupational physical activity throughout a work week, HCWs’ health may be improved, thus reducing absenteeism and increasing the sustainability of home care services. The two suggested redesign concepts should be considered areas for evaluation and adoption in practice by researchers and home care services in similar settings.

## Background

Home care workers (HCWs) in Norway provide nursing care and practical help for citizens who live at home but require assistance in daily living [1, 2]. Due to an aging population and a shift from hospital and institutional care to home-based health care, the need for HCWs in Norway, as well as in most Western societies, is increasing [3–5]. At the same time, HCWs find their work to be physically demanding and exhausting due to factors related to physical and psychosocial work demands created at the organizational level, as well as individual factors [6–10]. These aspects in combination may partly explain why the sick leave rates in the home care sector are nearly double the national Norwegian average, as well as the challenges to recruit and retain qualified HCWs [5, 8, 9, 11–13]. Thus, the HCWs’ working conditions need to improve to ensure continued provision of home care services.

Musculoskeletal pain is a leading cause of work disability among HCWs, with pain in the neck/shoulders and lower back areas being particularly prevalent [4, 14, 15]. While the causes of musculoskeletal pain are multifactorial, repetitive tasks, awkward postures, and heavy lifting, combined with individual factors such as low aerobic capacity may contribute to the large prevalence [4, 15–19].

In a recent observational study using accelerometers and a heart rate monitor to measure HCWs’ physical work demands in Norway, Tjøsvoll et al. [15] found that on average HCWs spent half of their workday sitting, whereas 37% and 12% were spent standing (including small movements) and being active (mainly walking), respectively. While this mean distribution is quite close to the 60(sitting):30(standing):10(active) split recommended by the European Agency for Safety and Health at Work [20], considerable deviations between individual workers were found, meaning that many HCWs were far from the recommended distribution. The study also observed large variations in arm elevation, forward trunk inclination, and work intensity between the HCWs [15]. Moreover, as HCWs’ heart rate rarely reaches sufficiently high levels (i.e., > 60% heart rate reserve) to improve their cardiorespiratory fitness, these occupational physical activity behaviors may contribute to the above-mentioned concerns [15, 21, 22].

Several authors have proposed interventions for reducing or preventing work-related musculoskeletal pain among health care workers [6, 23, 24]. However, HCWs have rarely been the sole focus of these investigations and intervention strategies have predominantly depended on individual motivation for behavior change and/or took time away from productive work (e.g., training/education in work/leisure time exercise, behavioral or ergonomic training/education) [6, 25, 26]. Firm evidence on how to promote HCWs’ musculoskeletal health is lacking, and existing interventions appear to even fall short in reaching all workers, particularly those who need interventions the most. The interventions may even reduce productivity (i.e. the number of clients HCWs can visit in a workday [27]). Altogether, this may explain why interventions have so far failed to provide convincing results.

The recently proposed Goldilocks Work principles [28, 29] has gained considerable interest in aiming at promoting workers’ health and capacity by ensuring that the balance between work and recovery is "just right", and that this balance does not rely on individual motivation or reduce productivity. This can be achieved by modifying *how* and/or *when* workers perform tasks or by introducing *new tasks* [28, 29]. Designing work tasks with a healthy level of physical demands can enhance worker capacity, prevent sick leave and reduce chronic diseases such as musculoskeletal pain [28]. Following this approach could be essential in addressing the negative health issues among HCWs. When (re)designing work to offer a "just right" balance, a participatory approach including important stakeholders needs to be implemented [29]. This has in large been missing in the development of interventions to improve HCWs’ health [25]. The feasibility of the Goldilocks Work principles has been examined in different occupational settings [30–33]. In a study of childcare workers, researchers developed ‘Goldilocks-games’ in collaboration with stakeholders. Playing these pedagogic games with the children increased workers’ time spent in moderate to vigorous physical activity [33]. Another study conducted workshops with brewery employees, who proposed five modifications to reduce standing and increase time with high-intensity physical activity. Feasibility testing of these modifications showed promising results regarding behavior change and health promotion among the workers [32]. Both studies aimed to implement the redesigns at an organizational level without reducing productivity, and the results are expected to be reported in future studies [34, 35]. However, interventions following the Goldilocks Work principles have not yet been explored for HCWs.

The aim of this study was to 1) gather suggestions from home care employees on suitable organizational (re)design concepts for promoting HCWs’ physical health and 2) have researchers and managers define actionable behavioral aims for the HCWs (i.e., describing those practical changes in HCWs’ occupational physical activity that the concepts could cause) for each proposed (re)design concept and evaluate them in the context of the Goldilocks Work principles.

## Methods

The study was carried out between March 2021 and June 2021 in accordance with the Declaration of Helsinki [36].

### Study design

**Fig. 1:**
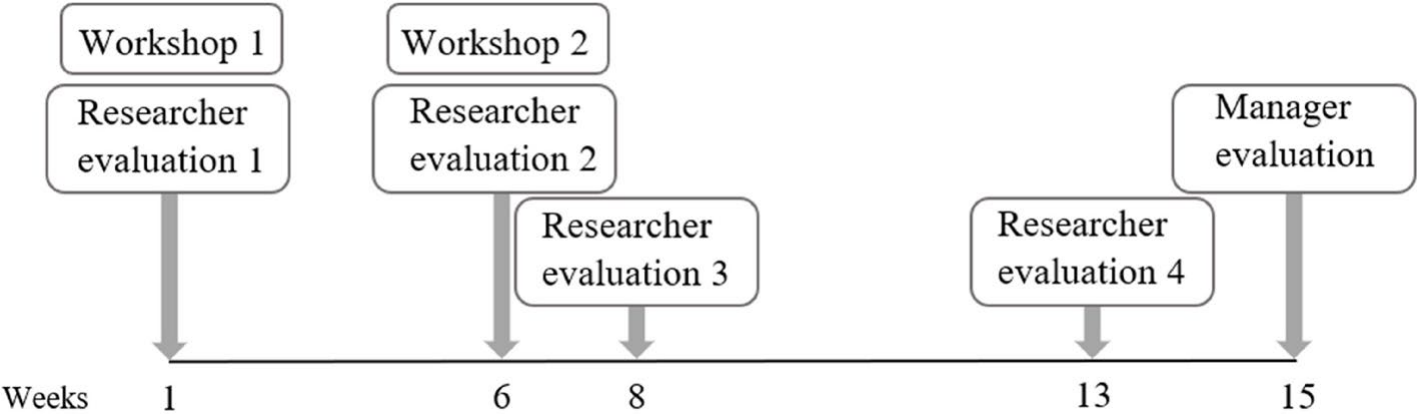
Study timeline. In weeks 1 and 6, Workshop 1 and Workshop 2 were conducted, followed by a researcher evaluation. In weeks 8 and 13, the researchers arranged additional evaluations guided by the literature on work and health, with the aim of formulating behavioral aims and evaluate the concepts based on the Goldilocks Work principles, which were then discussed with managers in week 15

The study utilized a participatory approach consisting of several steps (Fig. 1).

Two digital workshops were conducted involving home care employees, followed by assessments from researchers. Subsequently, two supplementary evaluation sessions were held with researchers prior to a joint evaluation with managers, who offered contextual understanding of the proposed (re)design concepts before agreement on the final evaluation. The study design separated the workshops and managerial evaluation to foster creativity and open-mindedness among both home care employees and managers.

### Research context

The Norwegian home care services are organized in municipalities [37]. Depending on the size of the municipality, the home care service is divided into smaller units based on geography. The current study was conducted in close collaboration with the home care services in Trondheim, which is the third largest municipality in Norway, with approximately 200,000 inhabitants. All thirteen home care units in the municipality were informed of the study and asked to express their interest in participating. Three units agreed to take part in the study. The three studied units (employing 60, 70, and 80 HCWs respectively) had a mean sickness absence of 14%, which is considerably above 11% and 7% cited for home care and all sectors nationwide [12, 13]. A majority of the HCWs in these units were assistant nurses with high school education, followed by nurses and other college/university health educated personnel (e.g., occupational therapists or welfare nurses), and assistant healthcare workers without formal education. HCWs’ work tasks included both direct and indirect care [3]. Direct care encompassed providing medical or practical assistance (i.e., assistance in hygiene, eating, dressing, and client transfers) [2, 11], whereas indirect care tasks incorporated documentation, administration, drug preparations, teaching and training tasks, and transportation [2, 3]. For all three studied units, work lists were generated manually by an operation coordinator (OC) in charge of determining which clients each HCW would visit at what times, as well as listing the work tasks to be performed during each visit, the duration of these tasks, and the transport mode (i.e., car or walk/bicycle/electronic scooter) to be used. Transportation mode provided by the unit depended on the geographical spread of the clients on HCWs’ work lists. The goal of such detailed planning was to ensure, wherever possible, good alignment between the assigned tasks and the HCWs’ training/education/competences. If the number of HCWs on shift exceeded the available work lists, the extra HCWs would be assigned office work for the whole shift (7.5 h). The HCWs were organized in smaller teams with a total of 20 − 25 members who could swap or help each other as required. As HCWs in the three studied units worked shifts (day, evening, and every third weekend), work lists rotated between HCWs within a team depending on who was at work in a particular shift.

### Digital workshops

#### Recruitment of home care employees

To explore the potential for (re)designing home care work according to the Goldilocks Work principles, important stakeholders in home care were identified in collaboration with unit managers. Thus, HCWs, safety representatives (also working as HCWs), and OCs working ≥ 50% in the three investigated units were invited to express their interest in participating in a two-hour digital workshop using Google Meet and the team collaboration software Miro [38]. Employees were informed of the study aims through an oral presentation by the first author and were required to complete a written informed consent form which was distributed by their unit managers. In order to include as many employees as possible that wanted to participate, workshop dates were set in collaboration between the first author and unit managers, and time was set aside during the day shift for employees wishing to take part. Two similar sessions were conducted, accommodating a total of 14 attendees, with characteristics shown in Table 1. Workshop 1 was attended to by eight participants: six OCs and two safety representatives who also worked as HCWs. In workshop 2, all six participants were HCWs.

**Table 1:** Characteristics of home care employees (*n* = 14) taking part in the two digital workshops

Characteristics	N (%)
**Current role**	**14**
Home care worker	6 (43)
Operation coordinator	6 (43)
Safety representative	2 (14)
**Formal education**	**12**^**a**^
Nurse	5 (42)
Assistant nurse	4 (33)
Occupational therapist	3 (25)
**Home care work experience**	**12**^**a**^
1 − 5 years	4 (33)
6 − 11 years	8 (67)
**Affiliation**	**14**
Unit 1	4 (28)
Unit 2	5 (36)
Unit 3	5 (36)
**Gender**	**14**
Male	7 (50)
Female	7 (50)

^a^ Missing data on two participants due to them sharing a computer with another participant and only one participant noting the requested information. The additional information (current role, affiliation and gender) was noted by the workshop leader during the plenum presentation

**Fig. 2:**
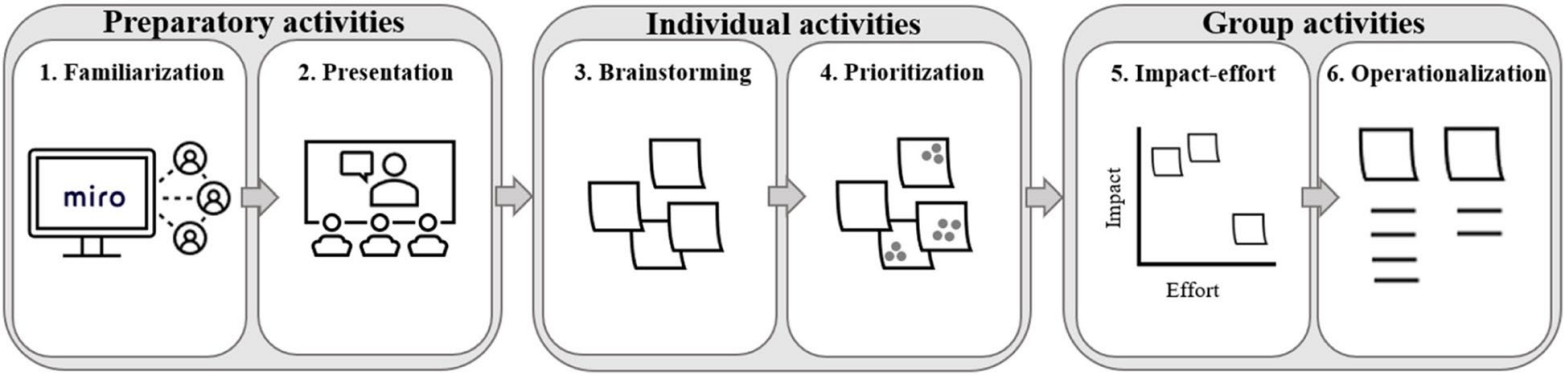
Digital workshop design. The first two steps were preparatory, focusing on creating a safe environment for idea generation, familiarizing the participants with the team collaboration software Miro, and providing the participants with relevant knowledge (e.g., regarding the Goldilocks Work principles and HCWs’ objectively measured occupational physical activity). The remaining steps included individual as well as group activities

Before the workshops, which were led by the first author (workshop leader), all participating home care employees were provided with an email containing links to Google Meet and the Miro workspace. The workshops followed a modified Lightning Decision Jam method providing a structured process for effective problem solving [39]. The workshop design is illustrated in Fig. 2 and described below.

#### 1. Familiarization

The goal of the first step was to familiarize the participants with the team collaboration software Miro and each other. Thus, the session commenced with a plenum introduction given by the workshop leader on how to use the software. Next, participants were asked to write virtual sticky notes containing their first name, work title/education, and duration of home care work experience. To create a more relaxed atmosphere for idea generation, participants were also asked to write one personal fun fact that had nothing to do with work. The same was done by the workshop leader, prompting other attendees to share their information with others. Any difficulties in operating the software were then addressed.

#### 2. Presentation

In this step, the workshop leader stated the workshop aim of gathering suggestions on suitable organizational (re)design concepts that could promote physical health among HCWs and gave a PowerPoint presentation of the Goldilocks Work approach. In the presentation, the workshop leader emphasized the aim of promoting workers’ health and capacity by ensuring that their physical workload and recovery periods are "just right". The workshop leader also informed on change methods that could be applied (i.e., changing how and/or when workers perform tasks and/or introducing new tasks), and the Goldilocks Work principles of interventions maintaining productivity and not being dependent on individual motivation when they are implemented (i.e., organizational-level implementation). The participants were also given practical examples of Goldilocks Work studies from the childcare and brewery sectors [32, 33]. Next, the workshop leader presented key results from Tjøsvoll et al.’s [15] study on musculoskeletal pain, aerobic capacity, and objectively measured occupational physical activity among HCWs from six home care units in the current municipality, including the three units in focus of the current investigation. Finally, the workshop leader explained the modified Lightning Decision Jam method [39], after which the participants were encouraged to ask any questions that had not been addressed during the presentations.

#### 3. Brainstorming

Based on the information provided in the preceding step, participants were given five minutes to write their ideas for possible Goldilocks Work (re)design concepts aimed at promoting HCWs’ physical health on sticky notes which were added to a collective virtual board.

#### 4. Prioritization

In this step, participants were required to prioritize between the suggested (re)design concepts. To facilitate this process, the workshop leader read all sticky notes out loud and consolidated similar ideas if participants agreed. Then, each participant was given six voting dots to place on the (re)design concepts they perceived to be the most feasible for their occupational context and have the highest potential for promoting health. The voting session was conducted individually on the collective board and lasted four minutes.

#### 5. Impact−effort evaluation

In this step, all (re)design concepts that had received at least three votes in the preceding step were placed on an impact − effort scale after plenum discussions and consensus among the participants. All occupational physical activity (re)design concepts situated on or above the mid-section of the impact axis were further developed and operationalized in the last workshop step.

#### 6. Operationalization

Finally, the occupational physical activity (re)design concepts perceived to have the highest potential impact on HCWs’ health were placed on a collective board. Participants were given the opportunity to elaborate on these (re)design concepts and operationalize them into concrete actions (e.g., possible implementers, practical aspects, and behavioral aims). The workshop leader wrote all opinions and concerns on the collective board.

### Evaluation process

The outcomes of the workshops underwent evaluation by researchers and subsequently by managers, with the intention of addressing questions related to health promotion potential, productivity impact, and dependency on individual motivation, as outlined in Table 2.

**Table 2:** Areas of evaluation including purpose and questions considered

Areas of evaluation	Purpose	Questions
**Potential for health promotion**	Formulate HCWs health-promoting behavioral aims for all proposed (re)design concepts	What were the workshop participants’ views on potential health effects of the redesign? How do we anticipate the (re)design to impact behaviors, and in what way? Is there a potential for the changes to promote physical health?
**Productivity**	Assess the (re)design concepts fulfillment of the Goldilocks Work principle of not reducing productivity	Which work tasks will be affected by the (re)design? Can the proposed (re)design concept potentially reduce productivity (i.e. the number of clients HCWs can visit in a workday [27])? If so, in what way and why? Are there ways to avoid any adverse effects on productivity if the concept is implemented?
**Independency of individual motivation**	Determine whether the concepts addressed the organizational level and thus were independent of individual motivation of HCWs	Is the proposed (re)design practiced at the organizational level? Will its implementation depend on HCWs’ individual motivation? Who should be responsible for implementing the (re)design?

All (re)design concepts that required either taking time away from productive work tasks or demanding individual motivation of HCWs to any extent would violate the Goldilocks Work principles. The two evaluation processes are described below.

#### 1. Researcher evaluation

The day after each workshop, authors IFL, MSF, and SR reviewed the written results in Miro and discussed initial thoughts related to the questions in Table 2. To support their evaluation, they subsequently reviewed relevant literature to determine whether similar (re)design concepts had been previously tested, what health and behavioral effects they seemed to provide, and whether indications were found of individual motivation being needed or a risk of impact on productivity. During the 8-week period between the researcher evaluation and the manager evaluation, the researchers met twice either digitally or physically to exchange further ideas and consolidate their findings.

#### 2. Manager evaluation

Manager evaluations were conducted in a 90-min Google meeting led by the first author IFL in which the three home care unit managers, as well as authors MSF and SR, participated. Characteristics of the managers are shown in Table 3.

**Table 3:** Characteristics of participants (*n* = 3) that took part in the managers evaluation

Characteristics	N (%)
**Formal education**	**3**
Nurse	3
**Affiliation**	**3**
Unit 1	1
Unit 2	1
Unit 3	1
**Gender**	3
Male	1
Female	2

First, IFL gave a PowerPoint presentation of the written information from the workshops in Miro and thoughts from the researcher evaluation. Next, based on their contextual insight, the managers were invited to share their opinions, as well as add information on the questions listed in Table 2. IFL wrote down the managers’ evaluations and gave a short plenum summary at the end of the meeting, in order to avoid misunderstandings and facilitate group consensus.

## Results

In the workshops, 39 sticky notes were created and ideas with similar contents were combined. After prioritization, ten ideas were placed on the impact-effort scale. Only those occupational physical activity (re)design concepts on or above the mid-section of the impact axis were further developed and operationalized. As shown in Table 4, the workshops resulted in five physical activity redesign concepts.

**Table 4:** Occupational physical activity redesign concepts proposed by home care employees

Redesign concepts	Workshop 1	Workshop 2
Operation coordinators should distribute work lists with different occupational physical activity demands more evenly between home care workers		
Operation coordinators should distribute transportation modes more evenly between home care workers		
Managers should facilitate correct use of ergonomic aids and techniques		
Home care workers should use the stairs instead of the elevator		
Home care workers should participate in home-based exercise training with clients		

: Proposed.  : Not proposed

A summary of the researcher evaluation (performed by authors IFL, MSF, and SR), as well as the manager evaluation (involving three home care unit managers) can be found in Table 5.

**Table 5:** Results of the researchers’ and managers’ formulations of behavioral aims and evaluation of Goldilocks Work principles fulfilment for the five redesign concepts

Redesign concepts	Behavioral aims	Maintain productivity	Independent of individual motivation
Operation coordinators should distribute work lists with different occupational physical activity demands more evenly between home care workers	Reducing inter-individual differences in occupational physical activity		
Operation coordinators should distribute transportation modes more evenly between home care workers	Reducing inter-individual differences in occupational physical activity		
Managers should facilitate correct use of ergonomic aids and techniques	Decrease time spent in awkward positions (trunk forward inclination and arm elevation)		
Home care workers should use the stairs instead of the elevator	Increase time dedicated to high-intensity physical activity at work		
Home care workers should participate in home-based exercise training with clients	Increase time available for strength and stretching exercises	/	

: Fulfilling the principle.  : Does not fulfill the principle.  /  : Fulfills the principle under some circumstances, not under other circumstances

The results from the workshops and evaluation process are described in more detail below.

### OCs should distribute work lists with different occupational physical activity demands more evenly between HCWs

This redesign concept could reduce inter-individual differences between HCWs in time spent sitting, standing, and being active, and with extensive trunk forward inclination and arm elevation; this was an issue raised by several workshop participants. Thus, they felt that lists containing many clients with low independence should be distributed more evenly among HCWs during a work week and should be interspersed with lists comprising less physically demanding clients. The concept was seen as highly beneficial for health among the HCWs, despite challenges due to varying skills, training, and difficulties in obtaining accurate information on both physiological and physically demanding tasks. Managers also emphasized issues that could further restrict alternation between lists, such as a need to limit the number of HCWs assisting a client per month, following national legislation, and minimizing transportation time. For implementation, the OC was identified as the key implementer, making the concept independent of individual worker motivation. Thus, researchers and managers agreed that if the mentioned concerns were taken into account, this concept fulfilled the Goldilocks Work principles.

### OCs should distribute transportation modes more evenly between HCWs

This redesign concept could reduce differences between HCWs in time spent sitting, standing, and being active and was motivated by the observation by several workshop participants that certain HCWs had access to a car more often than others. While the transportation HCWs had at their disposal depended on the geographical dispersion of clients on their work lists, participants felt that car allocation could be fairer and that a more equitable weekly schedule would be easy to implement. The transport alternations would have to be within a week and not during a day, as it would be unproductive to wait for someone to return with a car or a bicycle. However, some workshop participants expressed concern that planning HCWs to use active transportation against their will, could create psychosocial pressure for some. Additionally, suitable work clothes for Norwegian weather conditions were emphasized as necessary when using active transportation. The OCs was identified as implementers for this redesign which was viewed as one of two concepts having the highest potential impact on HCWs’ health. The redesign concept was found to fulfil the Goldilocks Work principles by researchers and managers as they believed that it would not affect the number of visits, and that individual motivation would not be an issue since the OCs were supposed to implement it.

### Managers should facilitate correct use of ergonomic aids and techniques

This redesign concept could decrease time HCWs spent in awkward positions (i.e., trunk forward inclination and arm elevation), and was proposed by the workshop participants as they were of view that incorrect and deficient use of ergonomic aids and techniques in clients’ homes contributed to the high levels of musculoskeletal pain among HCWs. Workshop participants rated the concept as requiring some effort to implement, as some individuals work primarily evenings or weekends, but would potentially have one of the highest impacts on HCWs’ health. The redesign concept was not perceived by researchers and managers to reduce productivity, although it would require some time set apart for educating and training the HCWs. However, its implementation would be dependent on HCWs’ individual motivation to use ergonomic aids and techniques in their daily work. Thus, the redesign concept would potentially only benefit the most motivated HCWs.

### HCWs should use the stairs instead of the elevator

This redesign concept could potentially increase the time HCWs spend performing high-intensity physical activity during their shifts, given that many of their clients live in tall apartment buildings. This was perceived as potentially having a high impact on HCWs’ health while incurring low implementation effort on behalf of their units. While both researchers and managers concurred that this redesign concept was not expected to reduce productivity much, if at all, the choice would ultimately depend on HCWs’ individual motivation and thus would not be in line with the Goldilocks Work principles.

### HCWs should participate in home-based exercise training with clients

This redesign concept could increase the time HCWs dedicated to strength and stretching exercises, and it was proposed by workshop participants, as HCWs are sometimes required to help/support clients in performing home exercises prescribed by physiotherapists or others. The workshop participants felt that this was an opportunity for the HCWs to serve as training partners for clients, thus increasing fitness while doing productive work. Consequently, participants were of view that this initiative would require low effort to implement, but also had the lowest potential impact on HCWs’ health, as HCWs would perform the same exercises as frail clients, and the fact that there were few such assignments in their units. Researcher and managers thought the concept could contributed to added time pressure if it prohibited HCWs from completing other assigned tasks while their clients’ performed exercises. Thus, by acting as training partners, they could be less productive. Moreover, both researchers and managers concurred that this redesign concept was dependent on individual motivation for behavior change and thus not in keeping with the Goldilocks Work principles.

## Discussion

The aim of this study was to 1) gather suggestions from home care employees on suitable organizational (re)design concepts for promoting HCWs’ physical health and 2) have researchers and managers define actionable behavioral aims for the HCWs for each proposed (re)design concept and evaluate them in the context of the Goldilocks Work principles. Two workshops held with the staff from three units resulted in five redesign concepts that HCWs, safety representatives, and OCs deemed beneficial for improving HCWs’ physical health. In the evaluation process, researchers and managers proposed behavioral aims for the five redesign concepts and evaluated whether they adversely impacted on productivity and would depend on the individual motivation of HCWs if implemented.

Three of the proposed redesign concepts were found to not fulfill the Goldilocks Work principles, namely having "managers facilitate correct use of ergonomic aids and techniques", having " HCWs use the stairs instead of the elevator" and having "HCWs participate in home-based exercise training with clients". Although similar interventions have been implemented and have yielded some benefits for HCWs [6, 23–25, 40], researchers and managers felt that their implementation would depend on individual worker motivation, which would in turn impact the overall effectiveness of the concept. For example, motivation is an important factor for successful implementation of patient handling interventions in healthcare [41] and interventions studies where the use of stairs was promoted by nudging techniques indicate that stair use decreases after the prompt is removed [42, 43]. Having HCWs participating in home-based strength and stretching exercises with clients is similar to an intervention implemented by Muramatsu et al. [40] where HCWs delivered a physical activity intervention to their clients. The results from the study indicates that individual motivation is an important factor for whether HCWs perform the exercises with their clients, as older HCWs (those aged ≥ 50 years) more often reported doing the program with the clients and that the program motivated them to be more physically active [40]. If implemented in a similar manner as in Muramatsu et al. [40], where the number of work tasks to be performed was increased, HCWs would likely spend more time with each client resulting in a reduction in the number of clients HCWs can visit within a shift. Therefore, the redesign concept could also be deemed detrimental to productivity. However, productivity in home care work can also include other aspects of quality of care [40, 44], which was not considered in this study.

The workshop participants also suggested having "OCs distributing work lists with different occupational physical activity demands more evenly between HCWs" with the view that this change would have a highly positive impact on HCWs’ health. This suggestion was evaluated by researchers and managers as independent of HCWs’ motivation and they felt that its implementation would not adversely impact productivity. Thus, guided by evidence (albeit inconsistent) indicating that too little variation in physical exposures could negatively affect musculoskeletal health [45, 46], especially if workers are required to spend long periods standing, in forward trunk inclination [47, 48], or with arms highly elevated [49], researchers and managers concurred that this intervention required further consideration. They were also motivated by the findings published by Czuba et al. [50], indicating that the percentage of time spent caring for clients with higher needs was associated with end-of-shift musculoskeletal pain and fatigue. Czuba et al. [50] suggested and pilot-tested a categorizing system for scheduling high-need clients more evenly between HCWs within workdays. However, the authors did not manage to accomplish their goal. Therefore, to avoid the same pitfalls, in the present study, a weekly rather than a daily schedule was subject to redesign, with the caveat that the effects of such interventions on musculoskeletal health are not sufficiently investigated [46], and the potential for predicting the outcomes is therefore limited.

The researchers and managers also concurred that having "OCs distributing transportation modes more evenly between HCWs" throughout a work week met the Goldilocks Work principles. Since time used on transportation in Norwegian home care accounts for 18 − 30% of total working time [3, 51], alternating between active and sedentary transportation would help alleviate musculoskeletal pain caused by spending prolonged periods in the same position [19]. These arguments are supported by the findings reported by Gerstel et al., who investigated the impact of replacing the use of private motorized vehicles among Swiss HCWs with walking, regular/electronic/foldable bicycles, public transportation, or a car-sharing system. In addition, the participants engaged in an educational physical exercise and nutrition program [52]. As the latter elements are dependent on individual motivation, they were not incorporated into the redesign concept proposed in the present study.

No previous study has evaluated a more even distribution of work lists with different occupational physical activity demands between HCWs throughout a work week. However, a cluster-randomized controlled study redistributing work schedules between HCWs to achieve a balanced weekly structure of physical work load, is being conducted in the municipality where the present investigation took place [53]. The aim is to evaluate the impact of the redesign on variation in physical activity and musculoskeletal health among HCWs. In addition, a feasibility study further developing the proposed redesign concept of distributing transportation modes more evenly between HCWs throughout a work week has been conducted in one of the current home care units and will be detailed elsewhere.

Some strengths and weaknesses should be acknowledged in the current study. First, a major strength is the strong focus on stakeholder involvement, which has previously been missing in the development of health promoting interventions for HCWs [25]. Engaging stakeholders in a participatory approach together with researchers increases the chances of identifying relevant Goldilocks Work redesign concepts that align with the home care context and can likely be realized in practice, while at the same time taking relevant research into account. However, while involving unit managers in identifying important stakeholders is beneficial, we acknowledge that other stakeholders exist, such as home care service clients. Identifying and including these stakeholders may offer additional perspectives [54]. Further, as the study took place during the COVID-19 pandemic, digital solutions had to be used to ensure that as many employees from different units were able to participate in the same workshops. Thus, even though the sample size was rather small, this study design has contributed to the local generalizability of the proposed redesign concepts, even if having to partake in group activities remotely may have made collaboration more challenging. Second, as the study was carried out in collaboration with three home care units in one urban Norwegian municipality, no inferences should be made with regard to other units or Norwegian municipalities, even though the organization of home care services across the country is similar with the differences mainly arising from the transportation modes used by HCWs. Third, one of the strengths of this study stems from basing the workshops on objectively measured occupational physical exposure data from the current home care municipality [15], since self-reported physical exposures are prone to bias [55, 56]. However, the Tjøsvoll et al., [15] study reports descriptive statistics at an overall level, which may mask variations between and within HCWs. Thus, we do not know to which extent inter-individual variability occur between HCWs or within HCWs. A final limitation arises from the failure to capture the complexity of musculoskeletal pain by taking other relevant information more into account, including age [6, 7], organizational constraints such as restricted hiring, and psychosocial pressure; all factors of which are suspected to influence work-related musculoskeletal pain among health care workers [6]. We also acknowledge that focusing solely on redesign concepts for physical work demands might unintentionally affect other factors, such as psychosocial conditions. We encourage future research to assess psychosocial aspects of these redesign concepts for a more comprehensive understanding of their potential impacts.

## Conclusion

By utilizing a participatory approach including important stakeholders in three urban Norwegian home care units, we identified and evaluated two redesign concepts that were in line with the Goldilocks Work principles. Operation coordinators had a key role in both, which involved a redistribution of work lists and transportation modes among HCWs which may lead to reduced inter-individual differences between HCWs in occupational physical activity throughout a work week. Thus, HCWs’ health may be improved. The two suggested redesign concepts should be considered areas for evaluation and adoption in practice by researchers and home care services in similar settings as they may in turn reduce absenteeism and increase the sustainability of home care services.

## Data Availability

The dataset used during the current study can be available from the corresponding author on reasonable request.

## Abbreviations

HCW: Home care worker
OC: Operation coordinator
